# The distant studio: a survey of design students’ experience with distance educational formats

**DOI:** 10.1007/s10798-022-09804-8

**Published:** 2023-01-06

**Authors:** Carlos Rosa, João Ferreira

**Affiliations:** IADE, Faculdade de Design, Tecnologia e Comunicação, UNIDCOM/IADE, Unidade de Investigação em Design e Comunicação, Universidade Europeia, Av. D. Carlos I, 4, 1200-649 Lisbon, Portugal

**Keywords:** Design education, Design studio, Distance education, Questionnaire

## Abstract

The paper presents the results of a survey (*n* = 279) conducted with the students of an undergraduate design course. The questionnaire inquired design students’ preferences regarding educational formats (distance, in-person, or combination of both); the questionnaire further explored the issue by comparing between four types of classes (project, drawing, theoretical-practical, and theoretical) and by establishing comparisons between the students’ enrolment year. The main results reveal that design students prefer in-person formats, and preference for in-person educational formats increases regarding *project-based* or *drawing* type of classes; what is more, preference for in-person educational formats is appears to be higher in second and third-year students than in first-year ones. These results have implications for design education: a direct transfer of the typical design educational format to a distance setting may be misguided and design education may require specific formats (distance or otherwise) to offer a satisfactory pedagogical experience to its students.

## Introduction

In March 2020, universities worldwide moved their entire educational programmes online. In most cases, the transition was complete within a couple of days; this was caused by the Covid-19 pandemic outbreak as the global situation was unclear and uncertain, and national governments across the world enforced restrictions that included mandates to move all academic activity to remote settings. While many universities experimented with blended learning models before, the majority had to adapt quickly to ensure classes would continue, exams were conducted, supervisory meetings were held, and all else that makes up every day academic life.

With the spring semester ended, we faced the aftermath of an intense experiment with distance education that spanned March 2020 until June 2021. As vaccination proceeded, most universities returned to normal (meaning presential classes). This return to the usual educational setting in higher-education institutions appears to be durable, but it is important not to let this moment pass without analysing what happened during these 16 months.

Distance learning is not a phenomenon contingent on the Covid-19 pandemic. Offers for Distance education have been growing significantly over the last decade; a 2018 report from the Education Department’s National Centre for Education Statistics (McFarland et al, 2018) showed that in the USA, while postsecondary enrolment fell in 2017, the number of students who took courses online grew by 5.7 per cent. These figures are consistent with a report (Seaman et al., 2018) from the Babson Survey Research Group (an organization that tracks online enrolment) showing that between 2012 and 2016 the percentage of online enrolment in universities increased 17.2 while overall enrolment decreased. At the same time, universities such as Stanford and Harvard University have made courses accessible online in areas such as computer science,^1^ engineering, and business.

Concerning design education, even before the pandemic there was a steady stream of research investigating how technological development allows for the possible application of, for instance, virtual-reality technology (Bernardo & Duarte, 2022, Elvezio et al, 2017, Teklemarian et al., 2014,) and distance formats (Aldoy & Evans, 2020) into higher-education design courses. These types of studies tend to be exploratory, but they demonstrate that the technology is developing fast and is increasingly easier to access. Furthermore, higher-education institutions (HEIs) do not seem ready to discard their investment on technological infrastructure. In a recent survey in the European Higher Education Area (Gaebel et al, 2021), most HEIs confirmed that they plan to enhance digital capacity and experiment with alternative ways of teaching beyond the Covid-19 crisis.

The data suggests that the phenomenon of distance education is independent of specific events like pandemics. The technological resources are available, and the educational community needs to study and understand this phenomenon. The philosophy of technology suggests that technology is not a force in itself; instead, its effects on everyday life are complex, and depend on the relationship between technological development and social interaction (Higgs et al., 2000). Advanced digital technology is not going away, which means we need to understand how it may (or may not) be used to provide the best possible educational experience to students.

In this paper, we contribute to this reflection with a study conducted in an undergraduate design course in Portugal. It is crucial to examine this issue from a design perspective because design education combines the teaching of both practical skills and theoretical expertise; while more established academic disciplines are founded on theory, abstract or conceptual knowledge, and decades of research, design is a recent academic discipline, with a tight relationship with practice and therefore its pedagogy is closer to vocational training.

In the context of higher education, vocational courses face a double challenge: they must meet the standards of what is expected of a university degree and still consider the needs and expectations of the professional world. In design education, students are expected to understand abstract knowledge (the theories, history, and culture of design) and demonstrate mastery of the practical skills, tools, and habits of mind necessary for accomplished design practice.

Distance education models may be harder to implement in design education since vocational training depends on one-to-one tutorial teaching. This means teachers and students share the same space, and learning is based on practical experience. Tutorial-based educational models are often labelled *signature pedagogies* (Shulman, 2005), meaning the specific format of education that “organize[s] the fundamental ways in which future practitioners are educated for their new professions.” (p.52). Signature pedagogies are idiosyncratic instruction formats that prepare students to become members of a profession. In law, for instance, the preferred pedagogical method is the ‘Socratic interaction’ (Mintz, 2006; Osborn, 2011), where the first year of law school, is based on a series of case dialogues between instructor and individual students. Another example of a signature pedagogy is medicine, where we recall the image of the senior physician followed in a hospital ward by a small group of novices, discussing and asking questions about the diagnosis of patients’ diseases.

The design education equivalent of the aforementioned Socratic dialogues in law and medical bedside conversations are the ‘design crits’ (Adams, 2016). Design crits are the centre of the student’s experience in the design studio educational setting [see Adams & Siddiqui (2016) for a review]. The term ‘crit’ originated in architecture education (Dinham, 1989) to describe the daily short, individual, and informal meetings between teacher and student in the design studio. The meetings often occur at the student’s desk when both teacher and student discuss the project at hand. These everyday conversations (Ferreira, 2018a) form the backbone of the design studio educational experience, in which students are expected to progressively build their knowledge with each project and with each teacher-student interaction.

These conversations–the design crits–take centre stage in design education. The underlying philosophy that supports this educational format is the *design studio*. In short, the design studio is two things: (a) it is the space where students work alongside their colleagues and under the guidance of a teacher; (b) it is also a unique educational format and a pedagogical idea of how the teaching/learning process of design should unfold. These two aspects are indivisible, that is, the teaching/learning process is predicated on the simultaneous presence in a shared space of teacher and students.

Presence is required to allow spontaneous conversations to occur, social interaction to unfold naturally, impromptu groups to form, and serendipity. Furthermore, several studies have shown that teacher-student interaction in the design studio is mediated by visual design representations (VDRs)^2^ meaning the models, drawings, or sketches that gradually shape a design concept or idea; Lawson (2005) succinctly describes VDRs as the “ways of representing design situations.” (p.293) and Goldschmidt (1991) argues that to design implies the ongoing ability to generate representations of a (still) non-existent artefact and concludes that the final goal “(…) of the process of designing is the production of visual representations of the designed entity with enough completion and coherence to allow its construction.” (p.125).

Visual Design Representations play a critical role in the design students’ pedagogical experience; like Ferreira (2018b) argues, during a design conversation, VDRs mediate the dialogue between teacher and student. In fact, the teacher-student interaction is directly influenced by the representations the student brings to the discussion in more ways than one. On the one hand, the students’ VDRs illustrate the stage of development of their project, but on the other hand, VDRs also serve as a record of the evolution of the students’ (design) thinking, which leads Ferreira to conclude that VDRs can make the project explicit and the design process understandable.

Design education may be challenging to translate into educational formats that do away with teachers’ and students’ simultaneous physical presence in the same space for long periods. There seems to be a built-in inconsistency between distance learning formats and the design studio. However, conventional lecture-style moments at specific milestones could also fit a distance format. Likewise, purely evaluative moments could also fit into a distance education model. Whatever the case, the recent experience with distance learning is a problematic issue for design education that requires careful study.

In this paper, we approached this problem from the student’s perspective. After an intense period, it was essential to capture what design students thought about their experience with distance learning. It is important to note that the entire undergraduate design course had to move online; this included all the studio-based classes and the laboratory and theoretical ones. The questionnaire that supports this study distinguishes between theoretical and studio classes.

The goal was to capture students’ thoughts and feelings as they were still fresh and vivid from their experiences. Students’ input is vital to inform future analysis and reflection so that this temporary pedagogical experience may provide valuable information for future considerations of distance education models in design. While the contribution of this study is mainly empirical, in the following section, we will summarise the most significant aspects and well-established ideas about distance education, as well as a summary of the principles of studio education, in order to frame the study results and provide a meaningful and contextualised discussion.

## Theoretical framework

### Distance education

In their highly influential book^3^ Simonson and his colleagues (2015) defined *distance education* as “institution-based, formal education where the learning group is separated, and where interactive telecommunications systems are used to connect learners, resources, and instructors” (p.6). This is the definition that has been adopted by the Encyclopaedia Britannica (Simonson & Berg, 2016). In a similar vein, the fourth edition of the Handbook of Distance Education, edited by Moore and Diehl (2019), defined *distance education* as “teaching and planned learning in which the teaching normally occurs in a different place from learning, requiring communication through technologies, as well as special institutional organization” (p.xii).

This well-established definition is helpful to frame our study because distance education, as defined above, is broad enough to function as the umbrella term that includes variations such as *hybrid* or *blended* learning, online learning, and so on.^4^ The term *distance education* allows us to avoid derailing the discussion into a technical debate about fine distinctions between specific formats, which would fall outside the scope of this paper. In other words, for our study, it is sufficient to define distance education as an educational format where the participants of the educational process are often in different physical spaces, and the process requires the aid of technology and the support of the institutional organization to set up.

Simonson’s book shows that while distance education has been growing exponentially, students continue to prefer traditional formats of education because “(…) they prefer meeting with the learning group and the instructor in the classroom, the lecture hall, the seminar room, or the laboratory. Students report that they value the presence of a learning group and that the informal interactions that occur before and after, and sometimes during, a formal class are valuable components of the total learning experience” (p. 5). This is the case even though a governmental meta-study (2009) in the United States “concluded that online learning students achieved better than traditional students because they tended to allocate more time to their studies.” (p.7). This means that effective learning results do not necessarily correlate with student satisfaction with an educational format.

Distance educational formats are generally considered as effective as in-person educational settings. Nevertheless, here we reach a key point: how is the effectiveness of learning being measured? What are the main criteria to evaluate if the teaching–learning process is successful? While wide-reaching meta-studies (Dean et al., 2001; Ni, 2013; Russell, 1999) concluded that there is no significant difference (in student achievement) between distance education and traditional classroom settings, there is still the need to understand what is being measured and what is considered efficient.

From these well-established publications, we gather that the emphasis seems to be on the *transmission of information*, which means success is measured by how effective a particular pedagogical format is in transmitting information to students. Simonson (2015), for instance, summarises his position by saying, “it is not the fact that instruction is delivered in a traditional, face-to-face environment or at a distance that predicts learning” (p.8) to argue then that “distance education can be as effective as any other category of instruction. Learning occurs and knowledge is retained.” (p.9) The same point is expressed by another influential figure, Richard Clark (),^5^ who published two widely cited meta-studies (in 1983 and 2012) where the author argues that “[t]he best current evidence is that media are mere vehicles that deliver instruction but do not influence student achievement any more than the truck that delivers our groceries causes changes in nutrition … only the content of the vehicle can influence achievement” (1983, p. 445)”.

It is clear from the above that the effectiveness of a distance education format is measured according to the success of information transmission; that is, an educational format is adequate if it delivers information as well as or better than a traditional (in-person) format of education. However, this is true only if we think of education as a means to deliver information; Clark’s image of the truck delivering instruction to students is an extraordinarily reductionist view of education.

This educational perspective is problematic for two reasons; firstly, it portrays students as passive receptors of knowledge instead of active constructors and participants in the learning process. Educational research has been accumulating in the direction of the view that knowledge is generated by human experience. For example, a central figure in constructivism, Piaget, (2001), proposed that cognitive structures are constructed by people during their actions within each social medium. Most constructivists, such as Lev Vygotsky, (1986), share this view but place greater emphasis than Piaget on the importance of the social medium in the learning process. Vygotsky studied how learning is fundamentally a complex social process, and learning does not result from an individual effort but is primarily the result of social activity. The crucial importance of the social dimension for learning was also emphasised in John Dewey’s, (1998) work, who argued that knowledge emerges when people experience situations that have meaning and importance.

Constructivist views of education contain the pitfall of *blank-slatism*, the idea that people are born as blank slates and biology does not influence on the learning process. This is a misconception, since the cognitive predisposition for learning is also a product of an evolutionary process; for example, work in linguistics has uncovered evidence that point to the presence of evolutionarily determined knowledge structures in human cognition that prepare the mind to acquire language (Chomsky, 1965; Pinker, 1994). More recent cognitive studies show (in line with the insights from original proponents of constructivist approaches to education) that *understanding* emerges when a learner makes personal meaningful connections between ideas, facts, concepts, or theories. This means that learning is more about *understanding* information than *receiving* it because what people understand is connected through rules, theories, narratives, or even pure logic. Without meaningful connections, information will not hold in long-term memory.^6^ To this point, in a recent review from the fields of educational and cognitive psychology, Kirschner and Hendrick, (2020) offer a roadmap of the most important discoveries in how learning happens; the authors recognise that novices learn differently than experts and, crucially, learning always requires a change in long-term memory.^7^

Students gain a deeper understanding of learning material when they construct meaning based on their experiences and interaction in the world (Krajcik et al., 2008) and there is more to learning than the transmission of information. Also, the *unidirectional transmission of information* view of education proposed by Simonson and Clark discards knowledge that is not easy to ‘transmit’, such as tacit or procedural forms of knowledge (Polanyi, 2005, 2009). These are experiential and embodied types of knowledge that require guided practice. Ericson’s^8^ research on ‘deliberate practice’ (Ericsson & Pole, 2016) shows that tutorial learning is a decisive factor in high-achieving learning of skills. Furthermore, several studies suggest (Astin, 1993; Chadwick & Ward, 1987; Chow, 2005; Yu & Kim, 2008) that students value other aspects beyond the transmission of information and attending university could be understood as a complex social and culturally enriching experience; this means that when students chose distance learning it may be a choice motivated by convenience, not preference.

If we assume that the delivery of instruction–or the transmission of information–is the goal of education, then distance education works as well as other formats of education. It seems evident that any educational experience also includes (at some point) the transmission of information. The critical issue is that information transmission and the delivery of instruction are not the *end goal* of education but a *means*. This seems particularly relevant in higher education. The American educational reformer John Dewey put the point succinctly when he argued that education is both a social process and a process of personal growth, and that education is not preparation for life but *is life itself*.^9^ Furthermore, as a person reaches adulthood and enters university, the challenge is to learn how to think about the world, not replicate the world.

University at its best should offer a well-rounded human experience. During a university course, students must engage with complex ideas, learn to argue and to reason through competing theories, and engage in careful discussions with teachers and peers about the material. Echoing Dewey, the American author and university teacher David Foster Wallace argued that being educated was fundamentally about understanding how to think, meaning “the real value of a real education, which has almost nothing to do with knowledge, and everything to do with simple awareness; awareness of what is so real and essential (…) it is unimaginably hard to do this, to stay conscious and alive in the adult world day in and day out. Which means yet another grand cliché turns out to be true: your education really IS the job of a lifetime.” (Wallace, 2009 unpaged).

In the case of higher education in design, the different perspectives on what the goal of education should be, are sharpened when comparing design with other academic disciplines. Most design courses believe that students must practice designing to learn how to design (Lawson, 2005) because design education is based on the learning-by-doing paradigm. It could be argued that it is unnecessary to be in the same physical space to practice a skill [even though that would contradict what is known about high-level expert practice (Ericsson & Poole, 2016)]; furthermore, the design studio educational setting has other specificities that may be incompatible with distance education principles. That being the case, what makes the design studio a unique education format then?

### Design studio

Regardless of curriculum, in a higher-education design degree, students spend most of their time in the design studio (Ibrahim & Utaberta, 2012), working on projects under the supervision of a teacher. As Lawson, (2004) noted in a global review of design schools: “All those schools of design understand this too and use methods of learning by doing in the ‘studio’ format as their primary educational tool.” (p.7). Studio experience is central to a student’s educational success since design schools adopt a learning-by-doing pedagogy. Learning how to design gradually unfolds as students tackle increasingly challenging design projects. Each project is a stepping stone towards design mastery.

The term design studio has two dimensions; it is both the physical space where students practice designing under the supervision of a teacher and the educational model that describes how the teaching and learning process of design should unfold. Cennamo and Brandt, (2012) summarised this point by saying that the design studio “is simultaneously, a class, a space, and a pedagogical method of instruction.” (p.840) both the space and the pedagogical ideas of the design studio contain features that set it apart. We cannot separate the uniqueness of the studio space from the educational ideas that inform it. The studio space sets the conditions for the pedagogical ideas to unfold; in other words, the space is a necessary feature that establishes the optimal conditions for students to learn how to design. Corazzo’s research specifically focuses on the studio space's influence for the students’ pedagogical experience (Corazzo, 2020), and he describes the studio space as “(…) a visually and materially unique learning space, and perhaps the pre-eminent ‘signature pedagogy’ of the creative disciplines” (Corazzo, 2019, p.1250), while Green, (2005), in similar lines, proposed a generic description of the studio’s configuration as “usually a large room equipped with drawing tables and chairs to enable students to work independently on projects.”(p.10).

In short, studio education requires limited class sizes, enough space for students to work, access outside scheduled classes, and constantly displaying the projects’ work in progress (via posters, models, prototypes). Also, regular studio sessions tend to be long and open-ended; design projects can take weeks to complete, which means students work on their projects, making steady progress from session to session; or otherwise wander the room, talk to each other, listen to music, and make the space their own (Austerlitz, 2007). Furthermore, the teacher often takes up a ‘coaching’ role (Goldschmidt, 2002), thus offering tutorial support from desk to desk. To this point, Boling and Smith, (2014) described the everyday role of studio teachers as:

The instructor spends the entire work period moving from one table to another confronting the problems that arise for each student designer as their projects take shape, and the critique period guiding discussion. Short, impromptu talks occur when a key principle comes up in the context of work, or when multiple students have reached a similar impasse or insight. (p.40).

The design studio is a complex educational setting where physical space and pedagogical approach are combined. In the studio, the interaction between teacher and student often transcends the appreciation or critique of the project at hand; like Dannels observed in a series of studies (Dannels, 2005; Dannels & Martin, 2008), the design studio is a social learning space where students gradually develop their personal designer identities by experiencing different types of oral communication. Experienced studio teachers appear aware of this (McDonnell, 2015, 2016; Yorgancioglu & Tunali, 2020) and adjust their feedback to address different aspects of the student’s ongoing development as a designer. In a succinct view, Goldschmidt et al., (2010) summarise the design studio educational setting as:

The studio is a working space, but also a group of students who undertake design exercises, or projects as they are usually called, typically during one semester at a time, under the guidance of teachers (…) who are experienced designers but only rarely expert educators. A studio class typically meets two or three times a week for several hours, during which students present and discuss their work in progress with their teachers and sometimes also with classmates and guests. (p.285).

The design studio is different from the usual university lecture room. A typical university lecture usually takes place in an auditorium where the teacher stands in front of an audience of seated students. The spatial arrangement suggests a different teaching and learning process: in a lecture hall, the teacher is transmitting information while the students listen, take notes, and ask questions. In this setting, knowledge is transmitted from teacher to students; the teachers expose what they know to the students, who then demonstrate they have apprehended the content by taking an exam. This is quite different from the studio teacher, who can wander from desk to desk, have short conversations and sit next to the students to conduct dialogues. Given the above, the structure of the design studio setting can be summarised as follows:

1. There is a fluid organisation of time and space;
2. Studio sessions are organised progressively according to the stages of the design project;
3. Feedback from the design teacher takes the form of open-ended one-on-one tutorials that lead to a final public presentation of the project to peers and faculty.

Point 3 is crucial because teacher-student tutorials constitute a potential moment when learning how to design occurs: in these ocasions, both teacher and student reflect and discuss the project, and in these interactions the student learns how to design as well as–crucially–how to think about *designing* (Cossentino, 2002; Farïas & Wilkie, 2016; and McDonald & Michela, 2019). These features support the central pedagogical purpose of the studio, which is the development of professional creative designers (Orr & Shreeve, 2018) who are able to self-reflect on their practice.^10^

## Study background

### Undergraduate design course context

This study was conducted at IADE–Faculty of Design, Technology and Communication; IADE is a private higher education institution based in Lisbon (Portugal) that offers degrees in Design, Communication, Marketing, Advertising, Computer Science and Engineering, and Photography. In addition, the design area of the faculty holds two undergraduate design courses,^11^ several Master’s, and a PhD in Design.

The study was conducted with students from the undergraduate design course. The course was established in 1969, making it the oldest design course in Portugal, and it is currently a large degree with about 530 students each year. An important point to remember is that these 500 + students are separated into smaller groups of about 25 to 30 students which remain stable throughout the entire course; it is typical for students to form friendships as the course unfolds. This structure allows students to spend three years sharing close experiences within a mostly stable group of colleagues; another benefit of organising students into smaller groups is that it allows teachers to get to know the students individually.

The undergraduate design course takes six semesters to complete and offers a total of 180 ECTS^12^; the course is structured to offer a wide array of experiences in different design disciplines, with each semester focusing on a particular domain (communication, product, multimedia, interaction, and environmental,) to provide a broad perspective of design that may lead to the pursuit of a specialised study in a Master’s degree.

Each semester includes a large project-based unit that occupies six hours per week (usually broken into two days holding three-hour sessions) and 12 ECTS. These units correspond to the design studio learning setting where students learn by tackling a complex design project under the supervision of a design teacher. This is the traditional setup of an undergraduate design course where studio time usually takes up more credits and contact hours. Finally, IADE (like other higher-education institutions specialising in design) offers access to facilities such as a print laboratory, 3D printing, photography studio, video editing studio, video-game development lab, and a dedicated library.

Finally, the course holds three types of course units: project-based (discussed above,) theoretical-practical (courses that combine both the teaching of theoretical ideas and the development of practical exercises,) and theoretical (purely lecture-based course units such as, for instance, Design History). In addition to these three types of course units, we categorised drawing units as the fourth category for this study. Officially, these units are considered project-based units, but they are distinct units that focus on developing drawing skills (sketching, observational drawing, technical, and analytical drawing). Thus, labelling them as a specific unit when inquiring the students about their educational experiences makes sense.

When the course moved into an online setting, the four types of classes were conducted via Blackboard Collaborate, a web conferencing platform that allows for synchronous audio and video conferencing. Teachers could still meet their classes synchronously and provide feedback to groups and individual students. Sessions were accessible through a web browser without the need for additional software. The platform permitted teachers and students to share their screens and upload and download files of their ongoing projects (in the case of students) and feedback (in the case of teachers). This setting ameliorated the difficulties raised by distance formats, particularly in the case of two-dimensional design (visual, communication, and multimedia design) but raised hurdles for three-dimensional design (such as product design).

## Methods

The study aimed to answer the following research questions:

1. Which educational format (in-person, hybrid, distance) do design students prefer?
2. Does this preference vary according to type of course unit (theoretical-practical, theoretical, project, or drawing)?

To do this, we designed an online questionnaire^13^ that was distributed via email. First, we gathered the email addresses of all the design students who were enrolled during the Covid-19 pandemic; this included first, second, and third-year students enrolled in the 2019–2020 and 2020–2021 academic years. The total number of students contacted was 794; we gathered 279 valid responses to the questionnaire, which means the sample corresponds to a significant 35% of our object of study (Table 1).

**Table 1:** Total number of responses

Number of students contacted	Number of responses	Percentage
794	279	35%

The students were contacted on 25 and 28 May of 2021, and the questionnaire was open until 28 June. After that, we downloaded the results into a spreadsheet, traced them for potential duplicates, and translated the results into English. The questionnaire was short (which may account for the high number of responses); we included a message prior to the survey’s questions which stated:

IMPORTANT NOTE: the questionnaire assumes that pandemics are cyclical but rare and exceptional. Therefore, please respond by considering a scenario where public health returned to normal.

This message was necessary because our aim was not to analyse the specific context caused by the Covid outbreak but the student experience with distance educational formats. The questionnaire contained the following types of questions:

1. Demographic data: age, sex, year of enrolment, nationality, and residency.
2. Preference of educational format; this section contain four equal questions corresponding to the four types of course units:

For project-based/drawing/theoretical-practical/theoretical curricular units which format do you prefer?

To answer, students had to select one of three options: in-person, remote, a combination of in-person and remote. There was a short paragraph reminding students which units corresponded to each category for each question. The questions in this section were central for the study because the answers compare student preference according to the course unit type, a critical issue we highlighted in the introduction.

Finally, we added three questions to complement the student’s input, which was:

How do you rate the following aspects of the daily experience at the campus: social interaction with colleagues, social interaction with teachers, and access to support infrastructures? (Likert scale answer).

As was mentioned, the answers regarding demographics and preference for educational format represent the core of this study. We analysed the data by aggregating the results and proceeded with a comparison of descriptive statistics. When we analysed the results of the four main questions of this study (the ones focused on the different educational formats), the outcome of the samples was not normally distributed. This meant we could use a nonparametric test to check some of the results of the comparison of the descriptive statistics. As such, the analysis was supported by a Wilcoxon signed ranked test and an Independent Samples Kruskal–Wallis test (statistical analysis of the study was done using SPSS software).

## Results

### Descriptive statistics comparison

#### Demographics

The study sample was N = 279 students; this figure corresponds to 35% of the students (794 in total) that experienced distance education during covid. This number of responses is, therefore, representative of the student body. In addition, most students were Portuguese (258), with only 21 international students. The demographical breakdown of the respondents is shown in the Table 2.

**Table 2:** Demographic Data

	Number of students	Percentage	Average age
Male	56	20%	23
Female	223	80%	22

The female to male skew is not a particular characteristic of this study’s sample; in fact, these results are consistent with the course’s average for the academic years of 18–19, 19–20, and 20–21, which was male (27%) and female (73%). Next, we present a breakdown of respondents by course year (Table 3).

**Table 3:** Number of respondents by enrolment year

First-year	Second-year	Third-year	No longer enrolled	Total
78	85	88	28	279

#### Answers

We begin by presenting the aggregate of all answers; the Table 4 shows the sum of student preferences regarding the type of classes (*in-person*, *remote*, or *combination* of both) for the four types of classes taken as a whole (project-based, theoretical-practical, theoretical, and drawing). The Table 4 provides an overview of overall student preference and serves as a baseline to compare with student preference for each class type, which we will address further ahead.

**Table 4:** Aggregate of student preference of type of class

Type of class	In person	Remote	Combination
Percentage	44,6%	25,6%	29,7%

As we can see from the Table 4, 44,6% of students reported preferring an in-person setting and 29,7% a combination of in-person and remote settings, whereas 25,6% stated that they prefer remote settings altogether. This overview provides a starting point for interpreting the results. However, when we break down the data by type of course unit and then by year of enrolment, we obtain a higher-resolution picture of student preferences.

#### Project-based course units

Students were asked what educational format they preferred regarding project-based course units (the design studio classes); the Table 5 reveals that a vast majority favour in-person (62%) or a combination of in-person and remote classes (32%), whereas only 6% mentioned distance setting. A breakdown of the data by enrolment year suggests a gap between the opinion of experienced students (second-year onwards) and novices (first-year).

**Table 5:** Student preference concerning Project-based course units

Question For project-based course units which format do you prefer?			
	In-person	Remote	Combination
1st-year	55%	10%	35%
2nd-year	60%	4%	36%
3rd-year	69%	6%	25%
No longer enrolled	64%	4%	32%
Total	62%	6%	32%

#### Theoretical-practical course units

Concerning theoretical-practical units, the students expressed a more balanced preference of educational format. When we analyse the data by year, we find some differences between novices and more experienced students; the most notable difference is the drop in preference for remote classes between first-year (36%) and second-year onwards (18%) (Table 6).

**Table 6:** Student preference concerning Theoretical-practical course units

Question For theoretical-practical course units which format do you prefer?			
	In-person	Remote	Combination
1st-year	31%	36%	33%
2nd-year	46%	18%	36%
3rd-year	31%	18%	51%
No longer enrolled	39%	18%	43%
Total	36%	23%	41%

#### Drawing units

For drawing units, the results were similar to project-based units; also, the figures decline regarding preference for remote settings (from 17 to 0%). In the case of students who are no longer enrolled, preference for in-person settings reaches 75% (Table 7).

**Table 7:** Student preference concerning Drawing course units

Question For drawing course units which format do you prefer?			
	In-person	Remote	Combination
1st-year	53%	17%	31%
2nd-year	62%	12%	26%
3rd-year	66%	9%	25%
No longer enrolled	75%	0%	25%
Total	62%	11%	27%

#### Theoretical course units

For theoretical classes, the student preference skews towards remote or combination settings; this result is different from the results of the other course units. Breaking the data into enrolment years suggests that first-year students are slightly more inclined to see the value in in-person teaching; however, this number falls as the years advance. Furthermore, 71% of second-year students prefer remote education formats for theoretical classes (this figure drops to 59% for third-year students) (Table 8).

**Table 8:** Student preference concerning Theoretical course units

Question For theoretical course units which format do you prefer?			
	In-person	Remote	Combination
1st-year	24%	59%	17%
2nd-year	14%	71%	15%
3rd-year	18%	59%	23%
No longer enrolled	14%	57%	29%
Total	18%	63%	19%

#### Everyday experience at campus

As a complement to the study's central questions, we asked students three questions about their everyday experience at the campus; the questions aimed to cover the general advantages of attending campus beyond the classes.

#### Social interaction with colleagues

To the question *how do you value (from 1—not at all important to 5—very important) social Interaction with colleagues*, the students gave the following answers (Table 9):

**Table 9:** Student appreciation of social interaction with colleagues on campus

Question How much do you value everyday social interaction with colleagues on campus?					
	1-not at all important	2-not important	3-neither important nor unimportant	4-important	5-very important
1st-year	0%	5%	19%	14%	62%
2nd-year	5%	1%	5%	26%	64%
3rd-year	2%	5%	11%	30%	52%
No longer enrolled	0%	14%	14%	25%	46%
Total	2%	5%	12%	24%	58%

The results show that, in general, most students (82%) value social interaction with colleagues on campus as *important* or *very important.* A breakdown by year of enrolment suggests a slight decline as the years progress, with students who finished the course reporting the highest frequency of *not important*.

#### Social interaction with teachers

To the question *how do you value (from 1—not at all important to 5—very important) Social Interaction with teachers*, the results show that 76% of students value interaction as either important or very important. The breakdown by year of enrolment suggests a slight decline as the years progress, with students who finished the course reporting the highest frequency of “2-not important”. Half the students report social interaction with teachers as “5-very important” (Table 10).

**Table 10:** Student appreciation of social interaction with teachers on campus

Question How much do you value everyday social interaction with teachers on campus?					
	1-not at all important	2-not important	3-neither important nor unimportant	4-important	5-very important
1st-year	1%	0%	27%	28%	44%
2nd-year	0%	0%	19%	26%	55%
3rd-year	3%	3%	17%	27%	49%
No longer enrolled	4%	18%	7%	14%	57%
Total	2%	5%	19%	26%	50%

#### *Access to support infrastructures (library, laboratories, study rooms, *etc.*)*

Most students (75%) rate access to support structures as either important or very important; this aspect is particularly valued by second-year students, where 67% classified the importance of access to infrastructures as 5-very important (Table 11).

**Table 11:** Student appreciation of support infrastructures on campus

Question How much do you value access to support infrastructures on campus?					
	1-not at all important	2-not important	3-neither important nor unimportant	4-important	5-very important
1st-year	3%	5%	21%	26%	46%
2nd-year	1%	1%	11%	20%	67%
3rd-year	7%	5%	23%	26%	40%
No longer enrolled	7%	7%	14%	36%	36%
Total	4%	4%	18%	25%	50%

### Nonparametric tests

The outcomes of the samples were not normally distributed, so we should use a nonparametric test to perform formal statistical analysis. For this study, it was important to check if there were significant differences between the answers of students enrolled in different course years (first, second, third year, and no longer enrolled) and to compare the results between the different educational formats (project, drawing, theoretical-practical, and theoretical).

To proceed, we considered the study data as ordinal, meaning a type of categorical data with an order. This means variables can be listed in an ordered manner and be numbered to indicate the order of the list. However, the list numbers are not measured but assigned as labels. This procedure allowed us to turn the answers (in person, remote, combination of both) into an ordered sequence of *in-person–combination–remote* where in-person and remote constitute the natural ends of the ordered list.

### Independent samples Kruskal–Wallis test

We used the Kruskal–Wallis test to determine whether there was a significant difference in distributions across the four years (first, second, third, and no longer enrolled). The null hypothesis is that the distribution is the same across the four year categories. We considered 0.05 to be the value to measure if the output is significant; any value equal to or below that threshold will be considered a statistically significant result.

The Kruskal–Wallis test showed no statistically significant difference in the educational preference score for each type of class between the four different enrolment years; the exception was the score for the Theoretical-practical classes (*p* = 0.048). We will elaborate on what these results mean in the Discussion section of this paper. The Table 12 presents a summary of the findings.

**Table 12:** Kruskal–Wallis test results

Independent samples Kruskal–Wallis test summary			
Educational format	Null hypothesis	Significance	Result
Project-based	The distribution of Project-based is the same across categories of *year*	.263	Retain the null hypothesis
Theoretical-practical	The distribution of Theoretical-practical is the same across categories of *year*	.048	Reject the null hypothesis
Drawing	The distribution of Drawing is the same across categories of *year*	.074	Retain the null hypothesis
Theoretical	The distribution of Theoretical is the same across categories of *year*	.324	Retain the null hypothesis

### Wilcoxon signed-rank test

The Wilcoxon signed-rank test is usually used to compare two related samples to test whether their mean ranks differ. In other words, the test is used to compare two sets of scores from the same participants, which helps investigate changes in scores from one variable to another. In the case of this study, the variables are the different types of classes (project-based, drawing, theoretical-practical, and theoretical).

For this test, the four types of classes were paired to test if there was a significant difference between the six pairs. The test showed a significant difference between all pairs except for the project-based–drawing pair (p = 0.172). The Table 13 presents a summary of the findings.

**Table 13:** Wilcoxon signed-rank test results

Wilcoxon signed rank-test summary			
Paired samples	Null hypothesis	Significance	Result
Project-based–theoretical	The median of differences between Project-based and Theoretical equals 0	< .001	Reject the null hypothesis
Project-based–drawing	The median of differences between Project-based and Drawing equals 0	.172	Retain the null hypothesis
Project-based–theoretical	The median of differences between Project-based and Theoretical-practical equals 0	< .001	Reject the null hypothesis
Drawing–theoretical	The median of differences between Project-based and Theoretical-practical equals 0	< .001	Reject the null hypothesis
Drawing–theoretical-practical	The median of differences between Project-based and Theoretical-practical equals 0	< .001	Reject the null hypothesis
Theoretical-practical–theoretical	The median of differences between Project-based and Theoretical-practical equals 0	< .001	Reject the null hypothesis

The Wilcoxon signed-rank test and the Independent Samples Kruskal–Wallis Test were necessary to clarify the insights drawn from the comparison of the descriptive statistics. The Kruskal–Wallis test revealed no statistically significant difference (except for theoretical-practical) in student preference across the years of enrolment in the course. This result does not support the descriptive statistics comparison where we noticed a pattern of student preference for in-person settings rising as the years advanced. A replication of this study or a research study focusing on this issue could clarify this inconsistency.

The Wilcoxon signed-rank test showed statistically significant differences in student preference according to the type of course unit (apart from the project-based–drawing pair). This means we can state with a high degree of confidence that if we compare different types of course units, we should find differences in student preference for educational format.

## Discussion

Our study aimed to capture design students’ perspectives after an intense experience with distance learning formats. The study was designed taking the specificity of design education into account, which means we acknowledged that the centre of the student’s educational experience is the design studio classes. As such, we aimed to answer two questions:

1. *Which educational format (in-person, remote, combination) do design students prefer?*
2. *Does this preference vary according to type of course unit (theoretical, theoretical-practical, drawing, or project)?*

Regarding these questions, we were not surprised to find that students prefer in-person educational settings (44, 6%) or a combination of in-person and remote (29, 7%), with 25, 6% expressing a preference for remote settings. This is consistent with what is known in the literature, that is, regardless of the type of higher-education course, when asked students state their first choice is not to learn at a distance but in-person educational settings (Simonson et al., 2015), whether it is a studio, a lecture hall, seminar room, or a laboratory.

This study supports the idea that students prefer synchronous in-person educational environments. The study further analysed this issue by comparing the results according to the type of course unit and the student’s year of enrolment. For project-based course units, most students prefer in-person settings; the preference appears to increase as the years progress, and experienced students value in-person design studio classes more (69%) than first-year students (55%). However, the rise in preference for in-person settings was not confirmed with the formal statistical test (the Independent Samples Kruskal–Wallis Test), which did not show the rise in preference as the years advanced to be statistically significant. More research is needed to clarify these results.

The difference in preference between novices and experienced students when comparing the descriptive statistics is consistent with previous studies on design education. For example, Donald Schön, (1987) described the peculiar predicament of the novice design student that “is expected to plunge into designing, trying from the very outset to do what he does not yet know how to do, in order to get the sort of experience that will help him learn what designing means.” (p. 93), in other words, novice design students do not know how to design before they start doing it themselves, so students are asked to design and learn how to design at the same time (Sachs, 1999).

As such, we can speculate that, as students mature and gain experience throughout a design course, they gradually grasp the implications of what it takes to learn how to design. For example, when first entering a design course, students may not be aware of the importance of studio classes’ long hours and open-endedness. Perhaps they could build this understanding as they experience designing from project to project. Another factor that might explain this apparent difference in preference is that the novices entered the course mid-Covid 19 pandemics. At the same time, senior students had a pre-Covid experience of the design course and could compare both pedagogical experiences (remote and in-person). The results of our study were not conclusive; more studies are needed that compare novices and senior design students, and notably lacking are longitudinal studies (following the same students across time).Design students prefer in-person settings for project-based (design studio) course units. This result is more apparent when we compare it with the results for other types of classes. For instance, for theoretical-practical units, students revealed a balanced distribution between in-person: 36%, remote 23%, or combination 41%, whereas, regarding drawing classes, the students expressed similar preferences with project-based ones, i.e., the preference is for in-person settings.

But the critical comparison to make is between theoretical and project-based classes. Design students report that they prefer theoretical classes in remote settings. Contrary to what we observed for project-based classes, the preference rises as the years advance. More first-year students (24%) prefer in-person classes for theoretical units than seniors (18%). This finding is important because it suggests that students do not appear to be biased towards in-person classes at the expense of distance educational formats; if that was the case, the result for different types of course units should be similar, and it was not.

The contrast in student preference—across all years—between theoretical and project-based classes suggests that having experienced what it was like not to have physical design studio classes, the students concluded that in-person classes were an essential factor in their educational experience. However, this insight is not conclusive. First, it is important to acknowledge the following limitation: the teachers and students had to replicate a course that was developed to be experienced in person in an online setting. A recent study (Bernardo & Duarte, 2020) revealed that design educators had trouble adapting (on such short notice) to the move from face-to-face to distance teaching. To reach a definite conclusion would require a study in which an undergraduate design course was explicitly developed to function in a distance format and then compare student satisfaction with students from traditional–in-person–courses. Nevertheless, our study's results suggest that design studio classes cannot (currently) be transferred to remote settings without considering that students prefer in-person settings for studio classes. It would also be unwise to ignore past and recent evidence (see, for instance, Ellmers, 2014; Adams & Sidiqui, 2016, Orr & Shreeve, 2018), suggesting that the design studio setting is an effective format for nurturing designers. The design studio is also a unique pedagogical setting within academia, a setting that, in theory, establishes the necessary conditions for a particularly creative kind of learning to occur.^14^ The problem, however, is that many of the studio’s main features and attributes such as participant proximity, synchronicity (the studio activities occur during specific and unrepeatable moments), and the pedagogical value of working together on the same project are difficult to make explicit (see Brandt (2013), Ferreira (2018a) and Jones (2021) for a careful elaboration on the implicit pedagogical elements of the design studio experience).

The design studio educational format was already questioned before the pandemic (Corazzo, 2019), but the centrality of the studio to design education was directly affected during the pandemic as universities forcibly adopted distance educational methods (Marshalsey & Sclater, 2020). Administrative pressure to increase student enrolment while decreasing the number of teachers^15^ is incompatible with an educational setting that depends on low numbers of undergraduates and long sessions per class. These features take up a large portion of space on campus and extended periods in already tight academic schedules. Also, it is common for studios to accommodate resources and technology such as specialised workshops and 2D and 3D laboratories that require specialised staff to operate. As a result, studio education is seen with suspicion by people outside its culture, particularly in a competitive and global Higher Education system that increasingly focuses on financial returns (Shreeve et al., 2010).

Finally, student preference does not necessarily correlate with the effectiveness of a pedagogical experience; in other words, what students prefer may not be the best setup for their education, even though student learning preferences are related to a predisposition to learn (Schmeck, 1983; Vermetten et al., 1999).

## Conclusions

This paper presented data on student preference for educational formats. The survey revealed that–in general–design students prefer in-person formats, and this overall inclination grows when students are asked about project-based or drawing type of classes. The results are clear; most students prefer in-person settings for those types of classes (usually associated with the design studio educational setting). The preference for in-person education formats appears to be higher in second and third-year students than in first-year ones, although this finding was not statistically significant.

Recent technological advances (such as generalised access to high-speed internet connectivity) mean distance education is more straightforward to implement than before; also, the Covid-19 pandemic forced universities worldwide to experiment with distance education whether they planned to or not. The impact of these measures on students’ and faculty’s educational experience is yet to be fully identified and analysed. The social and psychological impacts of measures targeted to mitigate the spread of Covid-19 will likely take years of research to understand. We cannot fully answer how the surrounding context of the pandemic influenced the questionnaire’s answers.

Nevertheless, we can postulate that the results would not be substantially different in a post-pandemic context, but that is a research question for another study. It is important to note that the results are particularly relevant to design education, and generalisations to other educational settings should be avoided or carefully conducted. Design courses' most important course units (in terms of allocated time and academic credits) are predicated on week upon week of long hours of project work conducted in the same room (the design studio setting). The design studio is a distinctive aspect of undergraduate design courses, which means that any present or future intervention in design education must consider the design studio format. Studies from other disciplines that follow a similar methodology to our study should carefully consider the implications of their pedagogies.

We suggest that if a design course were to move to a distance learning setting, it would have to be predicated on different educational foundations; a distance education offering in design would mean a new proposal of what a design course is and how it operates. A proportional view of this issue must include reflection on the possibility of a complementary approach that maintains the integrity of the design studio but complements it with specific approaches that are adequate to a design course.

From our study, the need to conduct further specific research emerges: On the one hand, it is necessary to understand the reasons that support student preference for in-person classes since these could be pedagogical (the students may feel in-person is the best format for their learning) or social (as suggested from our questionnaire, the students enjoy spending time at the campus) or other hitherto unknown reasons. On the other hand, a balanced interpretation of these results suggests that a design course developed specifically using distance education formats could generate higher levels of student satisfaction than the ones reported in this study. These questions must be answered before reaching definite conclusions regarding the adequacy or lack thereof of distance education formats for design education.

Finally, it is important to state that this study did not investigate the nuanced differences between types of distance learning formats; instead, the purpose was to obtain and analyse data on design students’ generic preferences regarding educational formats; the study’s results should be interpreted in that light.

## Data Availability

The complete database was submitted to *Data in Brief,* an open access and peer-reviewed journal, which publishes short, digestible articles that describe and provide access to research data (currently under review). Be that as it may, the authors declare that the database is fully available to any researchers who wish to use it in their studies by contacting the second author by email.

## Footnotes

1 Harvard University’s online course *Computer Science 50: Introduction to Computer Science I*, is entirely free and attracts hundreds of students every semester; the course has an established reputation (Orbey, 2020) as an example of how to offer a distance high-quality educational experience.

2 See the work of Pei, Campbell, and Evans (2011) For a description and taxonomy of visual design representations.

3 At the time of writing, the book is in its 7th edition.

4 According to Moore and Diehl (2019) in an online course at least 80% of content is delivered online; a blended or hybrid course combines online and face-to-face delivery, meaning 30% to 79% of the course’s content is delivered online; and finally, a web-facilitated course applies web-based technology to some extent but less than 30% of its content is delivered online.

5 Richard E. Clark is Emeritus Professor of Educational Psychology and Technology in the Rossier School of Education, and has published widely on educational research.

6 Regarding the importance of understanding for learning, an insightful study (Mueller & Oppenheimer 2014) measured and compared students who took notes either by hand or by typing on a laptop during a lecture; weeks later, the students who took notes by hand tested better, leading the authors to conclude that since handwriting is slower it forces the students to focus on the gist of the lecture, and not the details, but to note down the gist of a lecture the students had to understand it first.

7 Kirschner and Hendrick’s book offers a thorough analysis of the last decades’ key discoveries in how learning happens and explains their implications for educational practice; it offers a balanced and evidence-based perspective that overrides the short-sightedness of the traditional *nature-nurture* debates.

8 Anders Ericsson (1947–2020) was a Swedish psychologist and Professor of Psychology at Florida State University; he gained internationally recognition as a researcher in the psychological nature of expertise and human performance.

9 This idea is often presented as a quotation, when in fact it is a summary of several ideas that Dewey wrote in his seminal books My Pedagogic Creed (1897) and Democracy and Education (1916).

10 Donald Schön described this type of thinking as the capacity to consciously think about what one is doing while one is doing it and called it reflection-in-action (Schön, 1983).

11 The two undergraduate courses are the Design course and the Global Design course; the difference is that the Global Design course is exclusively taught in English and follows a Project-based Learning format.

12 The European Credit Transfer and Accumulation System (ECTS) is a tool to help students move between European Union countries and facilitate the recognition of their academic qualifications and study periods abroad.

13 We used the Google Forms platform to design and implement the questionnaire.

14 Interestingly, in a recent review of studies of the design studio (Boling, Schwier, Gray, Smith, and Campbell 2016), the authors describe the studio as an emerging mode of teaching and learning in different academic fields.

15 A recent study (Duncan, 2021) revealed that in the USA, a growing proportion of tuition is spent on administration rather than instruction.
